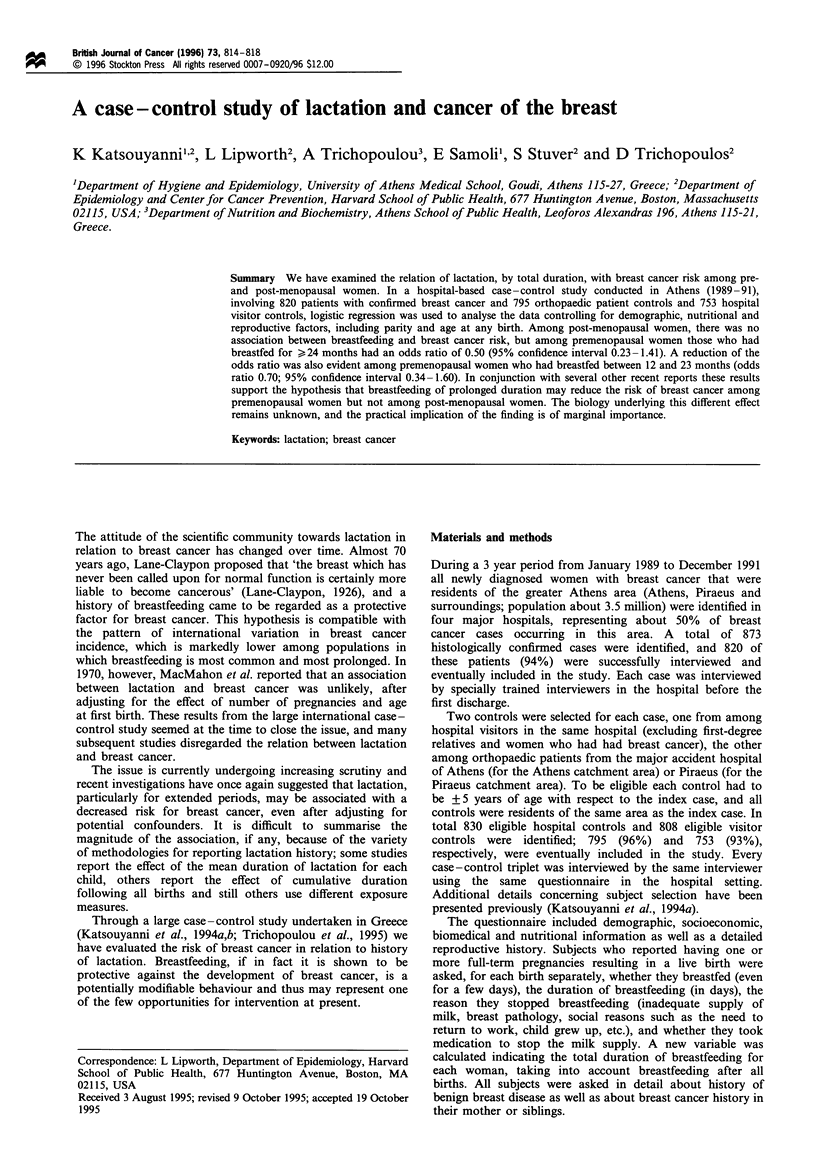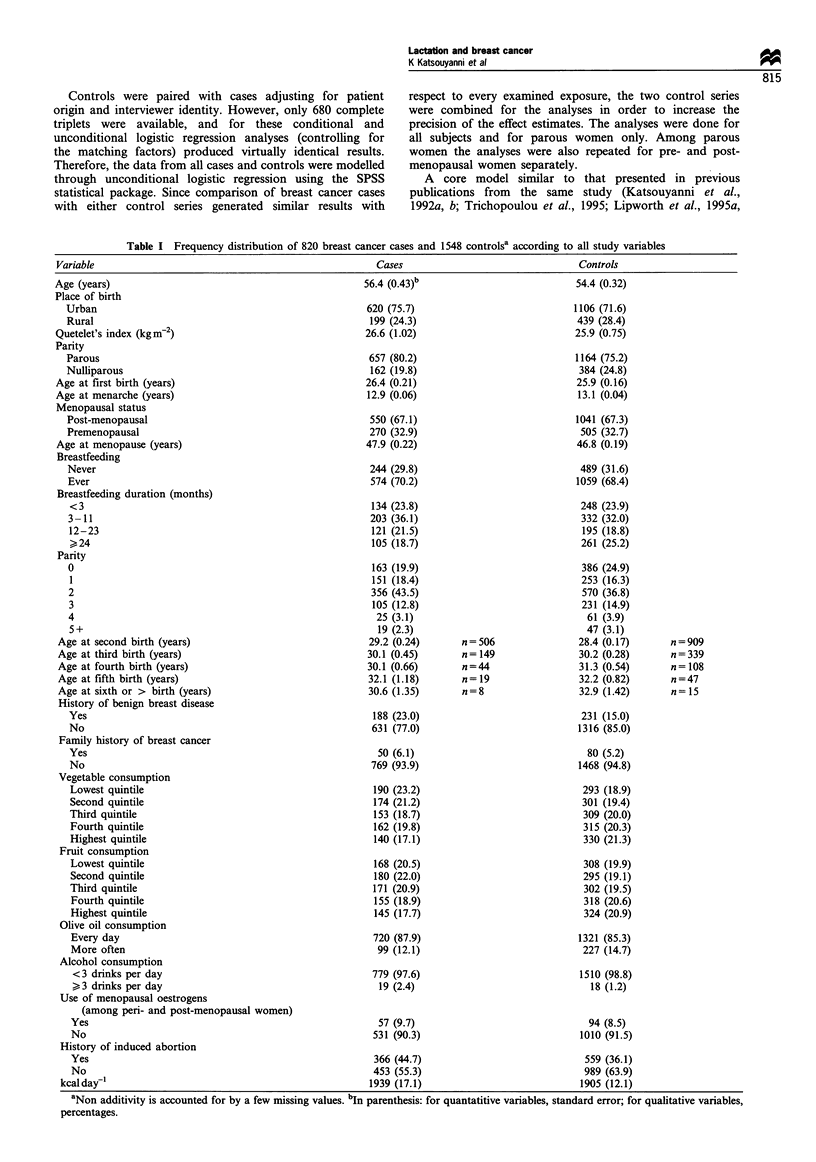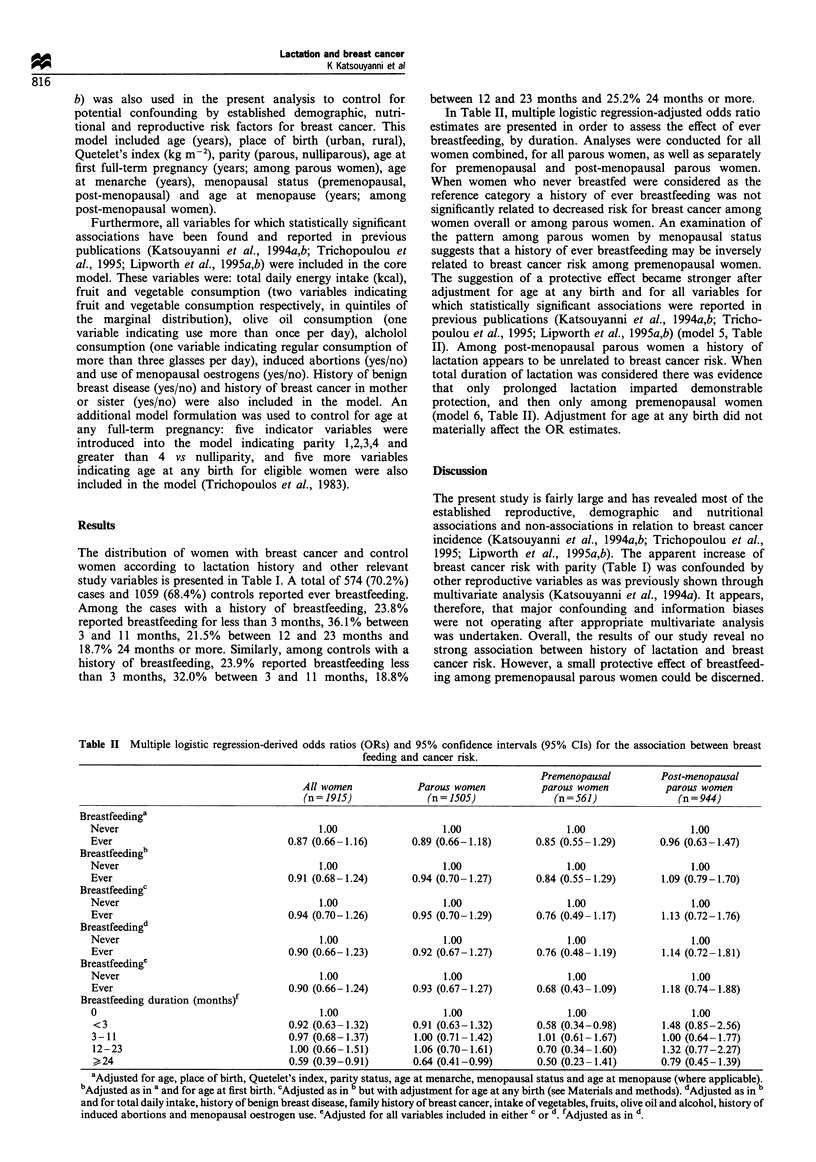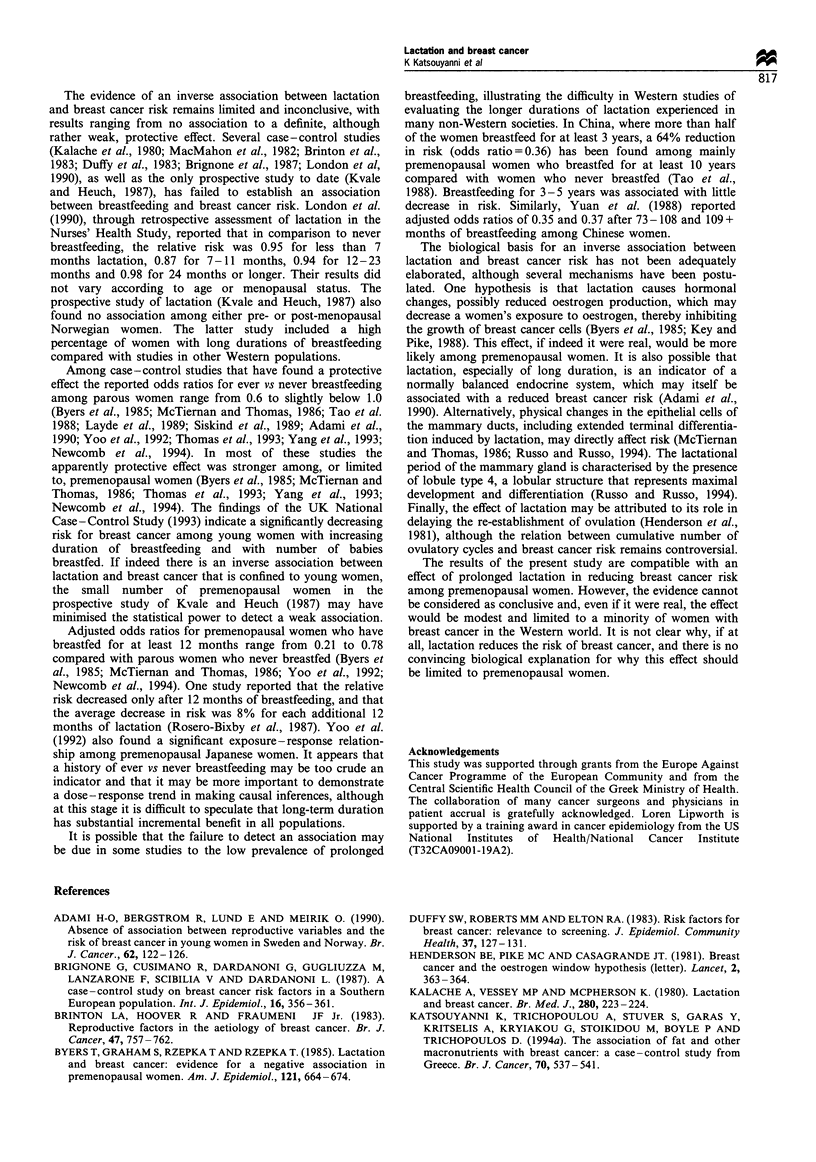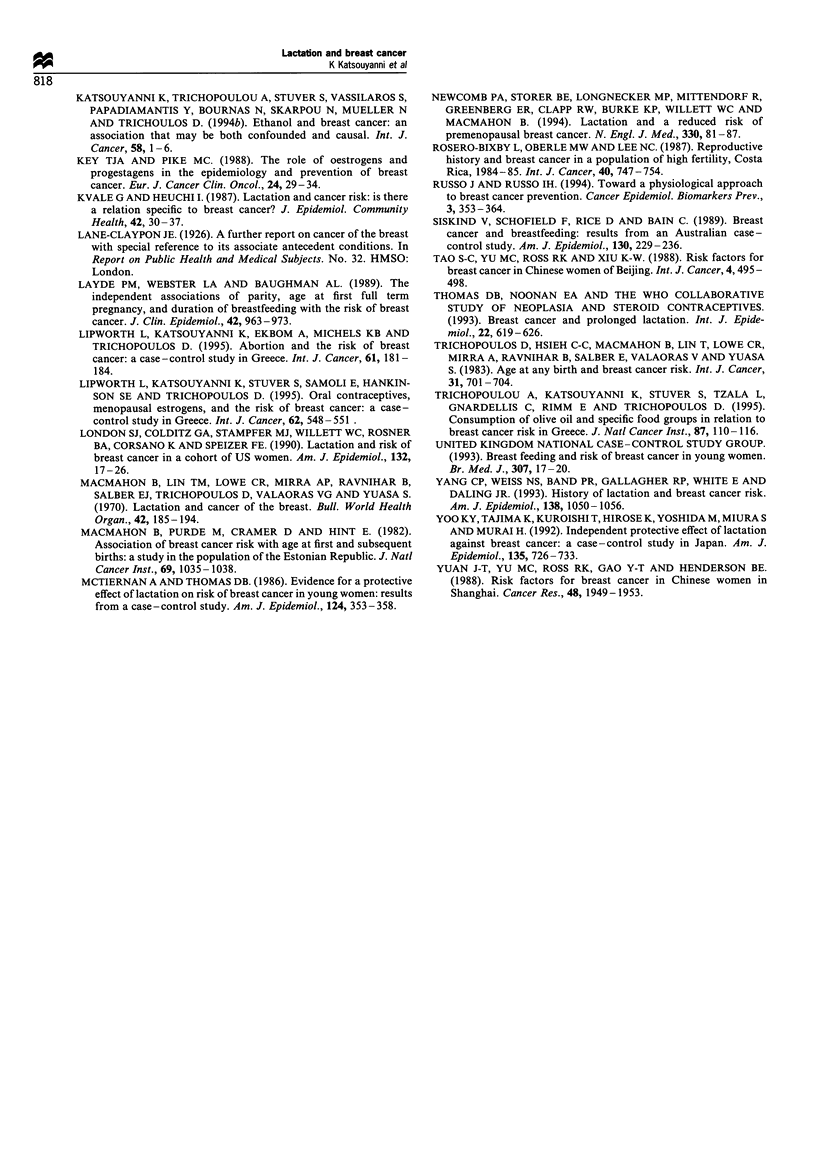# A case-control study of lactation and cancer of the breast.

**DOI:** 10.1038/bjc.1996.143

**Published:** 1996-03

**Authors:** K. Katsouyanni, L. Lipworth, A. Trichopoulou, E. Samoli, S. Stuver, D. Trichopoulos

**Affiliations:** Department of Hygiene and Epidemiology, University of Athens Medical School, Greece.

## Abstract

We have examined the relation of lactation, by total duration, with breast cancer risk among pre- and post-menopausal women. In a hospital-based case-control study conducted in Athens (1989-91), involving 820 patients with confirmed breast cancer and 795 orthopaedic patient controls and 753 hospital visitor controls, logistic regression was used to analyse the data controlling for demographic, nutritional and reproductive factors, including parity and age at any birth. Among post-menopausal women, there was no association between breastfeeding and breast cancer risk, but among premenopausal women those who has breastfed for > or = 24 months had an odds ratio of 0.50 (95% confidence interval 0.23-1.41). A reduction of the odds ration was also evident among premenopausal women who had breastfed between 12 and 23 months (odds ratio 0.70; 95% confidence interval 0.34-1.60). In conjunction with several other recent reports these results support the hypothesis that breastfeeding of prolonged duration may reduce the risk of breast cancer among premenopausal women but not among post-menopausal women. The biology underlying this different effect remains unknown, and the practical implication of the finding is a marginal importance.


British Journal of Cancer (1996) 73, 814–818
© 1996 Stockton Press All rights reserved 0007–0920/96 $12.00

### A case–control study of lactation and cancer of the breast

K Katsouyanni[1,2], L Lipworth[2], A Trichopoulou[3], E Samoli[1], S Stuver[2] and D Trichopoulos[2]

*[1]Department of Hygiene and Epidemiology, University of Athens Medical School, Goudi, Athens 115-27, Greece; [2]Department of Epidemiology and Center for Cancer Prevention, Harvard School of Public Health, 677 Huntington Avenue, Boston, Massachusetts 02115, USA; [3]Department of Nutrition and Biochemistry, Athens School of Public Health, Leoforos Alexandras 196, Athens 115-21, Greece.*

**Summary** We have examined the relation of lactation, by total duration, with breast cancer risk among pre- and post-menopausal women. In a hospital-based case–control study conducted in Athens (1989–91), involving 820 patients with confirmed breast cancer and 795 orthopaedic patient controls and 753 hospital visitor controls, logistic regression was used to analyse the data controlling for demographic, nutritional and reproductive factors, including parity and age at any birth. Among post-menopausal women, there was no association between breastfeeding and breast cancer risk, but among premenopausal women those who had breastfed for ⩾24 months had an odds ratio of 0.50 (95% confidence interval 0.23–1.41). A reduction of the odds ratio was also evident among premenopausal women who had breastfed between 12 and 23 months (odds ratio 0.70; 95% confidence interval 0.34–1.60). In conjunction with several other recent reports these results support the hypothesis that breastfeeding of prolonged duration may reduce the risk of breast cancer among premenopausal women but not among post-menopausal women. The biology underlying this different effect remains unknown, and the practical implication of the finding is of marginal importance.

**Keywords:** lactation; breast cancer

The attitude of the scientific community towards lactation in relation to breast cancer has changed over time. Almost 70 years ago, Lane-Claypon proposed that 'the breast which has never been called upon for normal function is certainly more liable to become cancerous' (Lane-Claypon, 1926), and a history of breastfeeding came to be regarded as a protective factor for breast cancer. This hypothesis is compatible with the pattern of international variation in breast cancer incidence, which is markedly lower among populations in which breastfeeding is most common and most prolonged. In 1970, however, MacMahon *et al.* reported that an association between lactation and breast cancer was unlikely, after adjusting for the effect of number of pregnancies and age at first birth. These results from the large international case–control study seemed at the time to close the issue, and many subsequent studies disregarded the relation between lactation and breast cancer.

The issue is currently undergoing increasing scrutiny and recent investigations have once again suggested that lactation, particularly for extended periods, may be associated with a decreased risk for breast cancer, even after adjusting for potential confounders. It is difficult to summarise the magnitude of the association, if any, because of the variety of methodologies for reporting lactation history; some studies report the effect of the mean duration of lactation for each child, others report the effect of cumulative duration following all births and still others use different exposure measures.

Through a large case–control study undertaken in Greece (Katsouyanni *et al.*, 1994*a*,*b*; Trichopoulou *et al.*, 1995) we have evaluated the risk of breast cancer in relation to history of lactation. Breastfeeding, if in fact it is shown to be protective against the development of breast cancer, is a potentially modifiable behaviour and thus may represent one of the few opportunities for intervention at present.

Correspondence: L Lipworth, Department of Epidemiology, Harvard School of Public Health, 677 Huntington Avenue, Boston, MA 02115, USA
Received 3 August 1995; revised 9 October 1995; accepted 19 October 1995

### Materials and methods

During a 3 year period from January 1989 to December 1991 all newly diagnosed women with breast cancer that were residents of the greater Athens area (Athens, Piraeus and surroundings; population about 3.5 million) were identified in four major hospitals, representing about 50% of breast cancer cases occurring in this area. A total of 873 histologically confirmed cases were identified, and 820 of these patients (94%) were successfully interviewed and eventually included in the study. Each case was interviewed by specially trained interviewers in the hospital before the first discharge.

Two controls were selected for each case, one from among hospital visitors in the same hospital (excluding first-degree relatives and women who had had breast cancer), the other among orthopaedic patients from the major accident hospital of Athens (for the Athens catchment area) or Piraeus (for the Piraeus catchment area). To be eligible each control had to be ±5 years of age with respect to the index case, and all controls were residents of the same area as the index case. In total 830 eligible hospital controls and 808 eligible visitor controls were identified; 795 (96%) and 753 (93%), respectively, were eventually included in the study. Every case–control triplet was interviewed by the same interviewer using the same questionnaire in the hospital setting. Additional details concerning subject selection have been presented previously (Katsouyanni *et al.*, 1994*a*).

The questionnaire included demographic, socioeconomic, biomedical and nutritional information as well as a detailed reproductive history. Subjects who reported having one or more full-term pregnancies resulting in a live birth were asked, for each birth separately, whether they breastfed (even for a few days), the duration of breastfeeding (in days), the reason they stopped breastfeeding (inadequate supply of milk, breast pathology, social reasons such as the need to return to work, child grew up, etc.), and whether they took medication to stop the milk supply. A new variable was calculated indicating the total duration of breastfeeding for each woman, taking into account breastfeeding after all births. All subjects were asked in detail about history of benign breast disease as well as about breast cancer history in their mother or siblings.

Controls were paired with cases adjusting for patient origin and interviewer identity. However, only 680 complete triplets were available, and for these conditional and unconditional logistic regression analyses (controlling for the matching factors) produced virtually identical results. Therefore, the data from all cases and controls were modelled through unconditional logistic regression using the SPSS statistical package. Since comparison of breast cancer cases with either control series generated similar results with respect to every examined exposure, the two control series were combined for the analyses in order to increase the precision of the effect estimates. The analyses were done for all subjects and for parous women only. Among parous women the analyses were also repeated for pre- and post-menopausal women separately.

A core model similar to that presented in previous publications from the same study (Katsouyanni *et al.*, 1992*a*, *b*; Trichopoulou *et al.*, 1995; Lipworth *et al.*, 1995*a*,

**Table I** Frequency distribution of 820 breast cancer cases and 1548 controls[a] according to all study variables

| *Variable* | *Cases* | | *Controls* | |
|---|---|---|---|---|
| Age (years) | 56.4 (0.43)[b] | | 54.4 (0.32) | |
| Place of birth | | | | |
| Urban | 620 (75.7) | | 1106 (71.6) | |
| Rural | 199 (24.3) | | 439 (28.4) | |
| Quetelet's index (kg m$^{-2}$) | 26.6 (1.02) | | 25.9 (0.75) | |
| Parity | | | | |
| Parous | 657 (80.2) | | 1164 (75.2) | |
| Nulliparous | 162 (19.8) | | 384 (24.8) | |
| Age at first birth (years) | 26.4 (0.21) | | 25.9 (0.16) | |
| Age at menarche (years) | 12.9 (0.06) | | 13.1 (0.04) | |
| Menopausal status | | | | |
| Post-menopausal | 550 (67.1) | | 1041 (67.3) | |
| Premenopausal | 270 (32.9) | | 505 (32.7) | |
| Age at menopause (years) | 47.9 (0.22) | | 46.8 (0.19) | |
| Breastfeeding | | | | |
| Never | 244 (29.8) | | 489 (31.6) | |
| Ever | 574 (70.2) | | 1059 (68.4) | |
| Breastfeeding duration (months) | | | | |
| <3 | 134 (23.8) | | 248 (23.9) | |
| 3–11 | 203 (36.1) | | 332 (32.0) | |
| 12–23 | 121 (21.5) | | 195 (18.8) | |
| ≥24 | 105 (18.7) | | 261 (25.2) | |
| Parity | | | | |
| 0 | 163 (19.9) | | 386 (24.9) | |
| 1 | 151 (18.4) | | 253 (16.3) | |
| 2 | 356 (43.5) | | 570 (36.8) | |
| 3 | 105 (12.8) | | 231 (14.9) | |
| 4 | 25 (3.1) | | 61 (3.9) | |
| 5+ | 19 (2.3) | | 47 (3.1) | |
| Age at second birth (years) | 29.2 (0.24) | $n=506$ | 28.4 (0.17) | $n=909$ |
| Age at third birth (years) | 30.1 (0.45) | $n=149$ | 30.2 (0.28) | $n=339$ |
| Age at fourth birth (years) | 30.1 (0.66) | $n=44$ | 31.3 (0.54) | $n=108$ |
| Age at fifth birth (years) | 32.1 (1.18) | $n=19$ | 32.2 (0.82) | $n=47$ |
| Age at sixth or > birth (years) | 30.6 (1.35) | $n=8$ | 32.9 (1.42) | $n=15$ |
| History of benign breast disease | | | | |
| Yes | 188 (23.0) | | 231 (15.0) | |
| No | 631 (77.0) | | 1316 (85.0) | |
| Family history of breast cancer | | | | |
| Yes | 50 (6.1) | | 80 (5.2) | |
| No | 769 (93.9) | | 1468 (94.8) | |
| Vegetable consumption | | | | |
| Lowest quintile | 190 (23.2) | | 293 (18.9) | |
| Second quintile | 174 (21.2) | | 301 (19.4) | |
| Third quintile | 153 (18.7) | | 309 (20.0) | |
| Fourth quintile | 162 (19.8) | | 315 (20.3) | |
| Highest quintile | 140 (17.1) | | 330 (21.3) | |
| Fruit consumption | | | | |
| Lowest quintile | 168 (20.5) | | 308 (19.9) | |
| Second quintile | 180 (22.0) | | 295 (19.1) | |
| Third quintile | 171 (20.9) | | 302 (19.5) | |
| Fourth quintile | 155 (18.9) | | 318 (20.6) | |
| Highest quintile | 145 (17.7) | | 324 (20.9) | |
| Olive oil consumption | | | | |
| Every day | 720 (87.9) | | 1321 (85.3) | |
| More often | 99 (12.1) | | 227 (14.7) | |
| Alcohol consumption | | | | |
| <3 drinks per day | 779 (97.6) | | 1510 (98.8) | |
| ≥3 drinks per day | 19 (2.4) | | 18 (1.2) | |
| Use of menopausal oestrogens (among peri- and post-menopausal women) | | | | |
| Yes | 57 (9.7) | | 94 (8.5) | |
| No | 531 (90.3) | | 1010 (91.5) | |
| History of induced abortion | | | | |
| Yes | 366 (44.7) | | 559 (36.1) | |
| No | 453 (55.3) | | 989 (63.9) | |
| kcal day$^{-1}$ | 1939 (17.1) | | 1905 (12.1) | |

[a]Non additivity is accounted for by a few missing values. [b]In parenthesis: for quantatitive variables, standard error; for qualitative variables, percentages.

b) was also used in the present analysis to control for potential confounding by established demographic, nutritional and reproductive risk factors for breast cancer. This model included age (years), place of birth (urban, rural), Quetelet's index (kg $m^{-2}$), parity (parous, nulliparous), age at first full-term pregnancy (years; among parous women), age at menarche (years), menopausal status (premenopausal, post-menopausal) and age at menopause (years; among post-menopausal women).

Furthermore, all variables for which statistically significant associations have been found and reported in previous publications (Katsouyanni *et al.*, 1994*a*,*b*; Trichopoulou *et al.*, 1995; Lipworth *et al.*, 1995*a*,*b*) were included in the core model. These variables were: total daily energy intake (kcal), fruit and vegetable consumption (two variables indicating fruit and vegetable consumption respectively, in quintiles of the marginal distribution), olive oil consumption (one variable indicating use more than once per day), alcholol consumption (one variable indicating regular consumption of more than three glasses per day), induced abortions (yes/no) and use of menopausal oestrogens (yes/no). History of benign breast disease (yes/no) and history of breast cancer in mother or sister (yes/no) were also included in the model. An additional model formulation was used to control for age at any full-term pregnancy: five indicator variables were introduced into the model indicating parity 1,2,3,4 and greater than 4 *vs* nulliparity, and five more variables indicating age at any birth for eligible women were also included in the model (Trichopoulos *et al.*, 1983).

### Results

The distribution of women with breast cancer and control women according to lactation history and other relevant study variables is presented in Table I. A total of 574 (70.2%) cases and 1059 (68.4%) controls reported ever breastfeeding. Among the cases with a history of breastfeeding, 23.8% reported breastfeeding for less than 3 months, 36.1% between 3 and 11 months, 21.5% between 12 and 23 months and 18.7% 24 months or more. Similarly, among controls with a history of breastfeeding, 23.9% reported breastfeeding less than 3 months, 32.0% between 3 and 11 months, 18.8% between 12 and 23 months and 25.2% 24 months or more.

In Table II, multiple logistic regression-adjusted odds ratio estimates are presented in order to assess the effect of ever breastfeeding, by duration. Analyses were conducted for all women combined, for all parous women, as well as separately for premenopausal and post-menopausal parous women. When women who never breastfed were considered as the reference category a history of ever breastfeeding was not significantly related to decreased risk for breast cancer among women overall or among parous women. An examination of the pattern among parous women by menopausal status suggests that a history of ever breastfeeding may be inversely related to breast cancer risk among premenopausal women. The suggestion of a protective effect became stronger after adjustment for age at any birth and for all variables for which statistically significant associations were reported in previous publications (Katsouyanni *et al.*, 1994*a*,*b*; Trichopoulou *et al.*, 1995; Lipworth *et al.*, 1995*a*,*b*) (model 5, Table II). Among post-menopausal parous women a history of lactation appears to be unrelated to breast cancer risk. When total duration of lactation was considered there was evidence that only prolonged lactation imparted demonstrable protection, and then only among premenopausal women (model 6, Table II). Adjustment for age at any birth did not materially affect the OR estimates.

### Discussion

The present study is fairly large and has revealed most of the established reproductive, demographic and nutritional associations and non-associations in relation to breast cancer incidence (Katsouyanni *et al.*, 1994*a*,*b*; Trichopoulou *et al.*, 1995; Lipworth *et al.*, 1995*a*,*b*). The apparent increase of breast cancer risk with parity (Table I) was confounded by other reproductive variables as was previously shown through multivariate analysis (Katsouyanni *et al.*, 1994*a*). It appears, therefore, that major confounding and information biases were not operating after appropriate multivariate analysis was undertaken. Overall, the results of our study reveal no strong association between history of lactation and breast cancer risk. However, a small protective effect of breastfeeding among premenopausal parous women could be discerned.

**Table II** Multiple logistic regression-derived odds ratios (ORs) and 95% confidence intervals (95% CIs) for the association between breast feeding and cancer risk.

| | *All women* (n=*1915*) | *Parous women* (n=*1505*) | *Premenopausal parous women* (n=*561*) | *Post-menopausal parous women* (n=*944*) |
|---|---|---|---|---|
| Breastfeeding[a] | | | | |
| Never | 1.00 | 1.00 | 1.00 | 1.00 |
| Ever | 0.87 (0.66–1.16) | 0.89 (0.66–1.18) | 0.85 (0.55–1.29) | 0.96 (0.63–1.47) |
| Breastfeeding[b] | | | | |
| Never | 1.00 | 1.00 | 1.00 | 1.00 |
| Ever | 0.91 (0.68–1.24) | 0.94 (0.70–1.27) | 0.84 (0.55–1.29) | 1.09 (0.79–1.70) |
| Breastfeeding[c] | | | | |
| Never | 1.00 | 1.00 | 1.00 | 1.00 |
| Ever | 0.94 (0.70–1.26) | 0.95 (0.70–1.29) | 0.76 (0.49–1.17) | 1.13 (0.72–1.76) |
| Breastfeeding[d] | | | | |
| Never | 1.00 | 1.00 | 1.00 | 1.00 |
| Ever | 0.90 (0.66–1.23) | 0.92 (0.67–1.27) | 0.76 (0.48–1.19) | 1.14 (0.72–1.81) |
| Breastfeeding[e] | | | | |
| Never | 1.00 | 1.00 | 1.00 | 1.00 |
| Ever | 0.90 (0.66–1.24) | 0.93 (0.67–1.27) | 0.68 (0.43–1.09) | 1.18 (0.74–1.88) |
| Breastfeeding duration (months)[f] | | | | |
| 0 | 1.00 | 1.00 | 1.00 | 1.00 |
| <3 | 0.92 (0.63–1.32) | 0.91 (0.63–1.32) | 0.58 (0.34–0.98) | 1.48 (0.85–2.56) |
| 3–11 | 0.97 (0.68–1.37) | 1.00 (0.71–1.42) | 1.01 (0.61–1.67) | 1.00 (0.64–1.77) |
| 12–23 | 1.00 (0.66–1.51) | 1.06 (0.70–1.61) | 0.70 (0.34–1.60) | 1.32 (0.77–2.27) |
| ⩾24 | 0.59 (0.39–0.91) | 0.64 (0.41–0.99) | 0.50 (0.23–1.41) | 0.79 (0.45–1.39) |

[a]Adjusted for age, place of birth, Quetelet's index, parity status, age at menarche, menopausal status and age at menopause (where applicable). [b]Adjusted as in [a] and for age at first birth. [c]Adjusted as in [b] but with adjustment for age at any birth (see Materials and methods). [d]Adjusted as in [b] and for total daily intake, history of benign breast disease, family history of breast cancer, intake of vegetables, fruits, olive oil and alcohol, history of induced abortions and menopausal oestrogen use. [e]Adjusted for all variables included in either [c] or [d]. [f]Adjusted as in [d].

The evidence of an inverse association between lactation and breast cancer risk remains limited and inconclusive, with results ranging from no association to a definite, although rather weak, protective effect. Several case–control studies (Kalache *et al*., 1980; MacMahon *et al*., 1982; Brinton *et al*., 1983; Duffy *et al*., 1983; Brignone *et al*., 1987; London *et al*, 1990), as well as the only prospective study to date (Kvale and Heuch, 1987), has failed to establish an association between breastfeeding and breast cancer risk. London *et al*. (1990), through retrospective assessment of lactation in the Nurses' Health Study, reported that in comparison to never breastfeeding, the relative risk was 0.95 for less than 7 months lactation, 0.87 for 7–11 months, 0.94 for 12–23 months and 0.98 for 24 months or longer. Their results did not vary according to age or menopausal status. The prospective study of lactation (Kvale and Heuch, 1987) also found no association among either pre- or post-menopausal Norwegian women. The latter study included a high percentage of women with long durations of breastfeeding compared with studies in other Western populations.

Among case–control studies that have found a protective effect the reported odds ratios for ever *vs* never breastfeeding among parous women range from 0.6 to slightly below 1.0 (Byers *et al*., 1985; McTiernan and Thomas, 1986; Tao *et al*. 1988; Layde *et al*., 1989; Siskind *et al*., 1989; Adami *et al*., 1990; Yoo *et al*., 1992; Thomas *et al*., 1993; Yang *et al*., 1993; Newcomb *et al*., 1994). In most of these studies the apparently protective effect was stronger among, or limited to, premenopausal women (Byers *et al*., 1985; McTiernan and Thomas, 1986; Thomas *et al*., 1993; Yang *et al*., 1993; Newcomb *et al*., 1994). The findings of the UK National Case–Control Study (1993) indicate a significantly decreasing risk for breast cancer among young women with increasing duration of breastfeeding and with number of babies breastfed. If indeed there is an inverse association between lactation and breast cancer that is confined to young women, the small number of premenopausal women in the prospective study of Kvale and Heuch (1987) may have minimised the statistical power to detect a weak association.

Adjusted odds ratios for premenopausal women who have breastfed for at least 12 months range from 0.21 to 0.78 compared with parous women who never breastfed (Byers *et al*., 1985; McTiernan and Thomas, 1986; Yoo *et al*., 1992; Newcomb *et al*., 1994). One study reported that the relative risk decreased only after 12 months of breastfeeding, and that the average decrease in risk was 8% for each additional 12 months of lactation (Rosero-Bixby *et al*., 1987). Yoo *et al*. (1992) also found a significant exposure–response relationship among premenopausal Japanese women. It appears that a history of ever *vs* never breastfeeding may be too crude an indicator and that it may be more important to demonstrate a dose–response trend in making causal inferences, although at this stage it is difficult to speculate that long-term duration has substantial incremental benefit in all populations.

It is possible that the failure to detect an association may be due in some studies to the low prevalence of prolonged breastfeeding, illustrating the difficulty in Western studies of evaluating the longer durations of lactation experienced in many non-Western societies. In China, where more than half of the women breastfeed for at least 3 years, a 64% reduction in risk (odds ratio = 0.36) has been found among mainly premenopausal women who breastfed for at least 10 years compared with women who never breastfed (Tao *et al*., 1988). Breastfeeding for 3–5 years was associated with little decrease in risk. Similarly, Yuan *et al*. (1988) reported adjusted odds ratios of 0.35 and 0.37 after 73–108 and 109+ months of breastfeeding among Chinese women.

The biological basis for an inverse association between lactation and breast cancer risk has not been adequately elaborated, although several mechanisms have been postulated. One hypothesis is that lactation causes hormonal changes, possibly reduced oestrogen production, which may decrease a women's exposure to oestrogen, thereby inhibiting the growth of breast cancer cells (Byers *et al*., 1985; Key and Pike, 1988). This effect, if indeed it were real, would be more likely among premenopausal women. It is also possible that lactation, especially of long duration, is an indicator of a normally balanced endocrine system, which may itself be associated with a reduced breast cancer risk (Adami *et al*., 1990). Alternatively, physical changes in the epithelial cells of the mammary ducts, including extended terminal differentiation induced by lactation, may directly affect risk (McTiernan and Thomas, 1986; Russo and Russo, 1994). The lactational period of the mammary gland is characterised by the presence of lobule type 4, a lobular structure that represents maximal development and differentiation (Russo and Russo, 1994). Finally, the effect of lactation may be attributed to its role in delaying the re-establishment of ovulation (Henderson *et al*., 1981), although the relation between cumulative number of ovulatory cycles and breast cancer risk remains controversial.

The results of the present study are compatible with an effect of prolonged lactation in reducing breast cancer risk among premenopausal women. However, the evidence cannot be considered as conclusive and, even if it were real, the effect would be modest and limited to a minority of women with breast cancer in the Western world. It is not clear why, if at all, lactation reduces the risk of breast cancer, and there is no convincing biological explanation for why this effect should be limited to premenopausal women.

**Acknowledgements**
This study was supported through grants from the Europe Against Cancer Programme of the European Community and from the Central Scientific Health Council of the Greek Ministry of Health. The collaboration of many cancer surgeons and physicians in patient accrual is gratefully acknowledged. Loren Lipworth is supported by a training award in cancer epidemiology from the US National Institutes of Health/National Cancer Institute (T32CA09001-19A2).

### References

ADAMI H-O, BERGSTROM R, LUND E AND MEIRIK O. (1990). Absence of association between reproductive variables and the risk of breast cancer in young women in Sweden and Norway. *Br. J. Cancer.*, **62**, 122–126.

BRIGNONE G, CUSIMANO R, DARDANONI G, GUGLIUZZA M, LANZARONE F, SCIBILIA V AND DARDANONI L. (1987). A case–control study on breast cancer risk factors in a Southern European population. *Int. J. Epidemiol.*, **16**, 356–361.

BRINTON LA, HOOVER R AND FRAUMENI JF Jr. (1983). Reproductive factors in the aetiology of breast cancer. *Br. J. Cancer*, **47**, 757–762.

BYERS T, GRAHAM S, RZEPKA T AND RZEPKA T. (1985). Lactation and breast cancer: evidence for a negative association in premenopausal women. *Am. J. Epidemiol.*, **121**, 664–674.

DUFFY SW, ROBERTS MM AND ELTON RA. (1983). Risk factors for breast cancer: relevance to screening. *J. Epidemiol. Community Health*, **37**, 127–131.

HENDERSON BE, PIKE MC AND CASAGRANDE JT. (1981). Breast cancer and the oestrogen window hypothesis (letter). *Lancet*, **2**, 363–364.

KALACHE A, VESSEY MP AND MCPHERSON K. (1980). Lactation and breast cancer. *Br. Med. J.*, **280**, 223–224.

KATSOUYANNI K, TRICHOPOULOU A, STUVER S, GARAS Y, KRITSELIS A, KRYIAKOU G, STOIKIDOU M, BOYLE P AND TRICHOPOULOS D. (1994*a*). The association of fat and other macronutrients with breast cancer: a case–control study from Greece. *Br. J. Cancer*, **70**, 537–541.

KATSOUYANNI K, TRICHOPOULOU A, STUVER S, VASSILAROS S, PAPADIAMANTIS Y, BOURNAS N, SKARPOU N, MUELLER N AND TRICHOULOS D. (1994*b*). Ethanol and breast cancer: an association that may be both confounded and causal. *Int. J. Cancer*, **58**, 1-6.

KEY TJA AND PIKE MC. (1988). The role of oestrogens and progestagens in the epidemiology and prevention of breast cancer. *Eur. J. Cancer Clin. Oncol.*, **24**, 29-34.

KVALE G AND HEUCHI I. (1987). Lactation and cancer risk: is there a relation specific to breast cancer? *J. Epidemiol. Community Health*, **42**, 30-37.

LANE-CLAYPON JE. (1926). A further report on cancer of the breast with special reference to its associate antecedent conditions. In *Report on Public Health and Medical Subjects*. No. 32. HMSO: London.

LAYDE PM, WEBSTER LA AND BAUGHMAN AL. (1989). The independent associations of parity, age at first full term pregnancy, and duration of breastfeeding with the risk of breast cancer. *J. Clin. Epidemiol.*, **42**, 963-973.

LIPWORTH L, KATSOUYANNI K, EKBOM A, MICHELS KB AND TRICHOPOULOS D. (1995). Abortion and the risk of breast cancer: a case-control study in Greece. *Int. J. Cancer*, **61**, 181-184.

LIPWORTH L, KATSOUYANNI K, STUVER S, SAMOLI E, HANKINSON SE AND TRICHOPOULOS D. (1995). Oral contraceptives, menopausal estrogens, and the risk of breast cancer: a case-control study in Greece. *Int. J. Cancer*, **62**, 548-551 .

LONDON SJ, COLDITZ GA, STAMPFER MJ, WILLETT WC, ROSNER BA, CORSANO K AND SPEIZER FE. (1990). Lactation and risk of breast cancer in a cohort of US women. *Am. J. Epidemiol.*, **132**, 17-26.

MACMAHON B, LIN TM, LOWE CR, MIRRA AP, RAVNIHAR B, SALBER EJ, TRICHOPOULOS D, VALAORAS VG AND YUASA S. (1970). Lactation and cancer of the breast. *Bull. World Health Organ.*, **42**, 185-194.

MACMAHON B, PURDE M, CRAMER D AND HINT E. (1982). Association of breast cancer risk with age at first and subsequent births: a study in the population of the Estonian Republic. *J. Natl Cancer Inst.*, **69**, 1035-1038.

MCTIERNAN A AND THOMAS DB. (1986). Evidence for a protective effect of lactation on risk of breast cancer in young women: results from a case-control study. *Am. J. Epidemiol.*, **124**, 353-358.

NEWCOMB PA, STORER BE, LONGNECKER MP, MITTENDORF R, GREENBERG ER, CLAPP RW, BURKE KP, WILLETT WC AND MACMAHON B. (1994). Lactation and a reduced risk of premenopausal breast cancer. *N. Engl. J. Med.*, **330**, 81-87.

ROSERO-BIXBY L, OBERLE MW AND LEE NC. (1987). Reproductive history and breast cancer in a population of high fertility, Costa Rica, 1984-85. *Int. J. Cancer*, **40**, 747-754.

RUSSO J AND RUSSO IH. (1994). Toward a physiological approach to breast cancer prevention. *Cancer Epidemiol. Biomarkers Prev.*, **3**, 353-364.

SISKIND V, SCHOFIELD F, RICE D AND BAIN C. (1989). Breast cancer and breastfeeding: results from an Australian case-control study. *Am. J. Epidemiol.*, **130**, 229-236.

TAO S-C, YU MC, ROSS RK AND XIU K-W. (1988). Risk factors for breast cancer in Chinese women of Beijing. *Int. J. Cancer*, **4**, 495-498.

THOMAS DB, NOONAN EA AND THE WHO COLLABORATIVE STUDY OF NEOPLASIA AND STEROID CONTRACEPTIVES. (1993). Breast cancer and prolonged lactation. *Int. J. Epidemiol.*, **22**, 619-626.

TRICHOPOULOS D, HSIEH C-C, MACMAHON B, LIN T, LOWE CR, MIRRA A, RAVNIHAR B, SALBER E, VALAORAS V AND YUASA S. (1983). Age at any birth and breast cancer risk. *Int. J. Cancer*, **31**, 701-704.

TRICHOPOULOU A, KATSOUYANNI K, STUVER S, TZALA L, GNARDELLIS C, RIMM E AND TRICHOPOULOS D. (1995). Consumption of olive oil and specific food groups in relation to breast cancer risk in Greece. *J. Natl Cancer Inst.*, **87**, 110-116.

UNITED KINGDOM NATIONAL CASE-CONTROL STUDY GROUP. (1993). Breast feeding and risk of breast cancer in young women. *Br. Med. J.*, **307**, 17-20.

YANG CP, WEISS NS, BAND PR, GALLAGHER RP, WHITE E AND DALING JR. (1993). History of lactation and breast cancer risk. *Am. J. Epidemiol.*, **138**, 1050-1056.

YOO KY, TAJIMA K, KUROISHI T, HIROSE K, YOSHIDA M, MIURA S AND MURAI H. (1992). Independent protective effect of lactation against breast cancer: a case-control study in Japan. *Am. J. Epidemiol.*, **135**, 726-733.

YUAN J-T, YU MC, ROSS RK, GAO Y-T AND HENDERSON BE. (1988). Risk factors for breast cancer in Chinese women in Shanghai. *Cancer Res.*, **48**, 1949-1953.